# Design and Fabrication of Double-Focused Ultrasound Transducers to Achieve Tight Focusing

**DOI:** 10.3390/s16081248

**Published:** 2016-08-06

**Authors:** Jihun Jang, Jin Ho Chang

**Affiliations:** 1 Department of Electronic Engineering, Sogang University, Seoul 04107, Korea; jhjang@sogang.ac.kr; 2 Department of Biomedical Engineering, Sogang University, Seoul 04107, Korea; 3 Sogang Institutes of Advanced Technology, Sogang University, Seoul 04107, Korea

**Keywords:** therapeutic ultrasound transducer, skin disease treatment, acoustic lens, press-focusing, double-focusing, 1-3 piezo-composite

## Abstract

Beauty treatment for skin requires a high-intensity focused ultrasound (HIFU) transducer to generate coagulative necrosis in a small focal volume (e.g., 1 mm^3^) placed at a shallow depth (3–4.5 mm from the skin surface). For this, it is desirable to make the F-number as small as possible under the largest possible aperture in order to generate ultrasound energy high enough to induce tissue coagulation in such a small focal volume. However, satisfying both conditions at the same time is demanding. To meet the requirements, this paper, therefore, proposes a double-focusing technique, in which the aperture of an ultrasound transducer is spherically shaped for initial focusing and an acoustic lens is used to finally focus ultrasound on a target depth of treatment; it is possible to achieve the F-number of unity or less while keeping the aperture of a transducer as large as possible. In accordance with the proposed method, we designed and fabricated a 7-MHz double-focused ultrasound transducer. The experimental results demonstrated that the fabricated double-focused transducer had a focal length of 10.2 mm reduced from an initial focal length of 15.2 mm and, thus, the F-number changed from 1.52 to 1.02. Based on the results, we concluded that the proposed double-focusing method is suitable to decrease F-number while maintaining a large aperture size.

## 1. Introduction

Light energy is prevalently used in beauty treatment for skin and surgery for cancers, such as melanoma. For this, photothermal therapy and photodynamic therapy (PDT) are currently available [1,2,3]. These methods are based on the absorption of incident light energy in a target. Since the light absorption in a specific target relies on its wavelength, an optimal wavelength should be selected in order to achieve high treatment efficacy. Note that an optimal wavelength is determined when a target highly absorbs the incident light energy with the wavelength. However, the optimal wavelength is hardly used because the light energy with the wavelength rarely reaches a target in many cases. This is mainly due to a limited penetration depth of light energy, so that a wavelength longer than an optimal one is generally used to increase penetration depth [4,5]. This causes lowering therapeutic efficacy that leads to inconsistent clinical results and multiple treatments [6].

High-intensity focused ultrasound (HIFU) has drawn many attentions as an alternative tool for skin treatment such as skin tightening [6,7,8,9]. For the skin treatment, high-intensity ultrasound (i.e., a source power of 0.45 to 7.6 J, [9]) should be delivered into either the dermis or the superficial muscular aponeurotic system (SMAS) to induce coagulative necrosis only in these layers without damage to the surrounding tissue and superficial layers. For this, a HIFU transducer should provide a small focal volume (e.g., 1 mm^3^) placed at a shallow depth (i.e., 3–4.5 mm from the skin surface) [6,9]. One advantage of HIFU surgery is that there is theoretically no limitation in selecting the location and the size of treatment region by determining the aperture size, focal depth, and operating frequency of a HIFU transducer. Unlike photothermal therapy and PDT, however, HIFU surgery does not have the capability for selective treatment: healthy tissues may be also irreversibly damaged if ultrasound energy fails to be focused on a desired treatment region. This is a particularly severe problem in skin treatment due to its small treatment region in the shallow depth. Real-time treatment monitoring by an imaging modality can be used to alleviate the risk in HIFU surgery [10,11]. At the same time, it is desirable that the focal volume is small enough to minimize deviation from a target.

At a given frequency, focusing becomes tight as the F-number, the ratio of focal length to aperture diameter, decreases. Several focusing schemes have been used for the purpose of HIFU surgery: either physical conformation or use of an acoustic lens for single element transducers and electronic focusing with either physical conformation or use of an acoustic lens for array transducers [12,13,14,15,16,17]. Note that single element transducers with either physical conformation or an acoustic lens is conventionally employed for skin treatment [14]. If a HIFU transducer needs to be in contact with the skin surface, the F-number should be larger than unity. This means that the aperture diameter of a HIFU transducer should be smaller than 4 mm for the purpose of skin treatment. With such an aperture, however, it is difficult to produce ultrasound intensity high enough to induce tissue coagulation. In addition, fabrication of an ultrasound transducer with the F-number less than unity is demanding; cracks frequently occur in the piezoelectric layer of a transducer, especially, a single crystal material during the fabrication process for press focusing in order to achieve a small F-number [12]. Therefore, the focal length of the general therapeutic transducers for skin treatment is much deeper than treatment depth to secure a large enough aperture; an additional medium such as water is placed in between the transducer and the skin surface to locate a focal point at a treatment area [13]. In particular, commercial therapeutic systems for skin treatment use a HIFU transducer immersed into a water-filled container, called a HIFU cartridge [14]. A practical problem of this method is the evaporation of the water due to high-intensity ultrasound, which requires a user to regularly refill a HIFU cartridge with water. This may cause the generation of air bubbles inside the cartridge, thus distorting an ultrasound beam field and deteriorating treatment efficacy. Additionally, high-intensity ultrasound may induce air bubbles inside a HIFU cartridge during and/or after treatment [18].

To overcome the limitation, in this paper, we propose a double-focused ultrasound transducer for which the transducer aperture is spherically shaped for initial focusing and an acoustic lens is used to finally focus ultrasound on a target depth. By doing so, it is possible to make a focal point come closer to a desired location without reducing aperture and changing the center of curvature in the physical conformation. This paper introduces the theoretical background and the design method for a double-focused ultrasound transducer. To verify the proposed concept, a 7.0 MHz double-focused ultrasound transducer with the F-number of unity was fabricated. Note that a center frequency of 7.0 MHz was selected because a commercial HIFU transducer for skin tightening has the center frequency. The press-focusing technique was used to fabricate a concave shaped transducer for initial focusing in this study. For easy press focusing, 1-3 piezo-composite was designed and fabricated as an active material. Furthermore, the following performances of the fabricated transducer were evaluated: pulse-echo response, beam profile, and electrical impedance.

## 2. Design and Fabrication of Double Focusing Transducers

### 2.1. Theoretical Background and Design

Ultrasound can be geometrically focused using two different methods: physical conformation and an acoustic lens [12,13,14,15,16]. For physical conformation, the aperture is spherically shaped so that ultrasound signals from the edge and the center of a piezoelectric material can simultaneously arrive at the center of curvature. In the case of an acoustic lens, also, ultrasound signals from the edge and the center of a piezoelectric material should arrive at a focal point at the same time after traveling through different lens thicknesses. Based on this assumption, we will derive equations to obtain design parameters for a lens.

**Figure 1:**
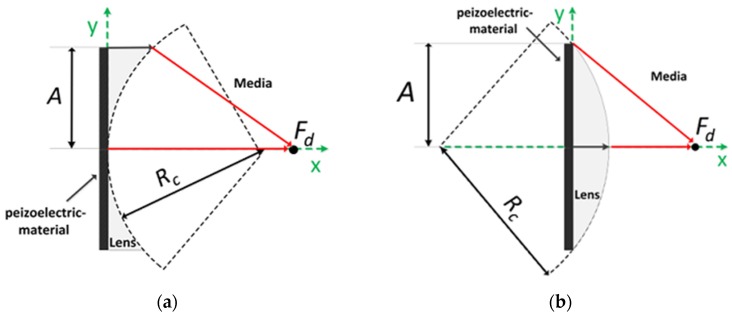
Conventional lens configuration and design parameters: (**a**) concave and (**b**) convex lenses.

Acoustic lens focusing uses sound speed variation in different media; the speed of sound of a lens material, *V_L_*, is either slower or faster than that of the human tissue, *V_M_*. If *V_L_* is slower than *V_M_*, an acoustic lens is of convex shape. At a given focal length, *F_d_*, the radius of curvature, *R_c_*, can be determined when it is satisfied that:

(1) $$\frac{\sqrt{A^{2}+F_{d}^{2}}}{V_{M}}=\frac{F_{d}-R_{c}+\sqrt{R_{c}^{2}-A^{2}}}{V_{M}}+\frac{R_{c}-\sqrt{R_{c}^{2}-A^{2}}}{V_{L}}$$

where *A* is the radius of aperture (see Figure 1). On the other hand, if *V_L_* is faster than *V_M_*, an acoustic lens becomes concave shape and Equation (1) changes to:

(2) $$\frac{F_{d}}{V_{M}}=\frac{\sqrt{A^{2}+{\left(F_{d}-R_{c}+\sqrt{R_{c}^{2}-A^{2}}\right)}^{2}}}{V_{M}}+\frac{R_{c}-\sqrt{R_{c}^{2}-A^{2}}}{V_{L}}$$

Note that the first-order Taylor series approximation of the square root functions in Equations (1) and (2) yields the well-known equations for *R_c_* of an acoustic lens, i.e., Rc=Fd(VMVL−1) and Rc=Fd(1−VMVL), respectively [16].

**Figure 2:**
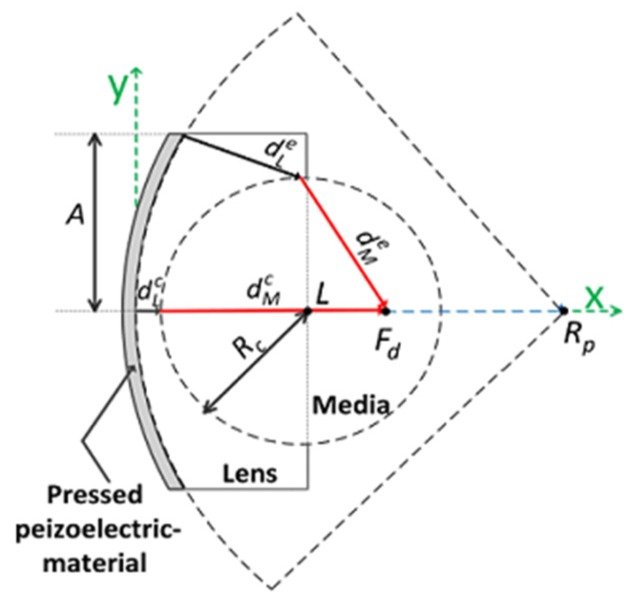
Schematic diagram to determine the radius of lens curvature for the proposed double-focused transducer.

In the design of a double-focused ultrasound transducer, it is necessary to determine an initial focal length for physical conformation and the radius of curvature for an acoustic lens determining a desired focal length as shown in Figure 2. The basic concept to determine the radius of lens curvature is equal to that for Equations (1) and (2), except that the aperture of a double-focused ultrasound transducer is of concave shape. In order to focus ultrasound energy at a desired focal point, the traveling time of ultrasound generated from the edge of an aperture should be equal to that from the center. This can be expressed as:

(3) $$\left|\left(\frac{d_{L}^{e}}{V_{L}}+\frac{d_{M}^{e}}{V_{M}}\right)-\left(\frac{d_{L}^{c}}{V_{L}}+\frac{d_{M}^{c}}{V_{M}}\right)\right|=0$$

where $d_{L}^{e}$ and $d_{M}^{e}$ are the traveling distance of ultrasound from the edge aperture through the lens material and human tissue, respectively. Note that the diffraction and the refraction of ultrasound are ignored for the simple design of a spherical lens. $d_{L}^{c}$ and $d_{M}^{c}$ are the traveling distance of ultrasound from the center aperture. From Figure 2, it is found that those traveling distances are given as:

(4) $$d_{L}^{e}=\sqrt{{\left(R_{p}-\sqrt{{R_{p}}^{2}-A^{2}}-\alpha \right)}^{2}+(A-\sqrt{{R_{c}}^{2}-{\left(\alpha -L\right)}^{2}}}$$

(5) $$d_{M}^{e}=\sqrt{{\left(\alpha -F_{d}\right)}^{2}+({R_{c}}^{2}-{\left(\alpha -L\right)}^{2})}$$

(6) $$d_{L}^{c}=L-R_{c}$$

(7) $$d_{M}^{c}=F_{d}-L+R_{c}$$

where:

(8) $$\alpha =\left(\left(L+\beta R_{p}\right)-\sqrt{{\left(L+\beta R_{p}\right)}^{2}-\left(1+\beta \right)\left(L^{2}+\beta {R_{p}}^{2}-{R_{c}}^{2}\right)}\right)/\left(1+\beta \right)$$

(9) $$\beta =A^{2}/({R_{p}}^{2}-A^{2})$$

and *L* is the maximum thickness of an acoustic Lens. *R_p_* and *R_c_* are the radius of curvature for physical conformation of a piezoelectric material and for a lens to conduct the desired focusing as illustrated in Figure 2.

**Figure 3:**
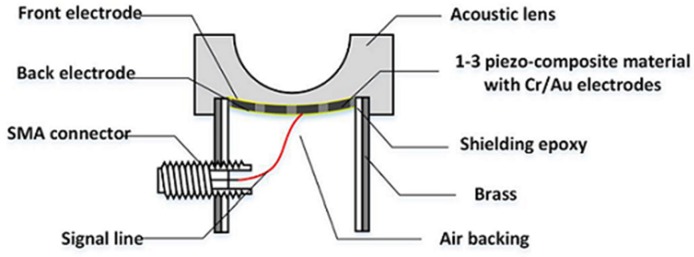
Conceptual illustration of a double-focused transducer in a cross-sectional view.

Substituting Equations (4)–(7) into Equation (3) yields:

(10) $$\left|\left(\begin{array}{c} \frac{\sqrt{{\left(R_{p}-\sqrt{{R_{p}}^{2}-A^{2}}-\alpha \right)}^{2}+(A-\sqrt{{R_{c}}^{2}-{\left(\alpha -L\right)}^{2}}}}{V_{L}}\\ +\frac{\sqrt{{\left(\alpha -F_{d}\right)}^{2}+({R_{c}}^{2}-{\left(\alpha -L\right)}^{2})}}{V_{M}} \end{array}\right)-\left(\frac{L-R_{c}}{V_{L}}+\frac{F_{d}-L+R_{c}}{V_{M}}\right)\right|=0$$

and the desired radius of curvature for a lens, *R_c_*, is determined by solving Equation (10). After selecting all parameters, except *R_c_*, the recursive computation of Equation (10) can be performed using MATLAB (Mathworks Inc., Natick, MA, USA) to find *R_c_* satisfying Equation (10). In this study, Epo-tek 301 (Epoxy technologies, Billerica, MA, USA) was selected as an acoustic lens material for a double-focused ultrasound transducer. The geometric parameters and sound velocities of the media used for the design of the double-focused transducer are summarized in Table 1. As a result, the recursive computation yielded *R_c_* of 6 mm. Figure 3 illustrates the cross-sectional view of a double-focused transducer designed for skin disease therapy. Here, the minimum lens thickness was computed to be 500 μm at the center of the aperture and the maximum lens thickness was 670 μm at the edge of the aperture from Equations (4) and (6).

**Figure 4:**
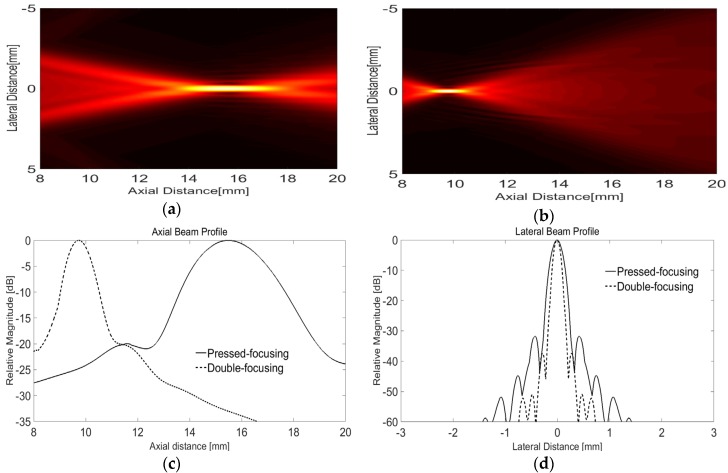
Beam propagation simulated using PZFlex: one-way radiation patterns produced by the concave transducer for initial focusing (**a**) and the double-focused transducer designed in this study (**b**). (**c**,**d**) show the lateral and axial beam profiles at the maximum intensity of the radiation patterns.

With the parameters summarized in Table 1, the double-focusing effect was examined by finite element method (FEM), i.e., PZFlex (Weidlinger Associates, Los Altos, CA, USA) simulation; the aperture and initial focal depth of a 7-MHz transducer were determined to 10 and 15 mm, respectively, and the radius of lens curvature was calculated to be 6 mm. As an active layer, we used a simple concave-shaped pressure load, i.e., a PLOD function in PZFlex, which enabled us to examine the sole effect of the acoustic lens on focusing performance. The concave shaped active layer was realized by using an axisymmetric simulation option. An air-backing block with a thickness of 5 mm and Epo-tek 301 as a lens material were utilized. The material properties of the lens material can be found in Table 2. A medium was water and absorbing boundary condition was assumed in order to avoid ultrasound reflection from each boundary. In the simulation, 1,054,920 square-shaped numerical elements with a width of 14.3 µm were used. According to our design, the lens should make it possible to reduce the focal length by 5 mm corresponding to the F-number of 1.0 (10/10) while the physical conformation led to the F-number of 1.5 (15/10). The focal point is placed at a depth of 3.5 mm from the skin surface because the lens plays another role of a standoff of which maximum thickness is 6.5 mm. Note that it can be assumed that ultrasound generated by a piezoelectric material hardly passes through the standoff. As shown in Figure 4a,b, the focal length of the double-focused transducer was 9.72 mm while that of the concave transducer was 15.49 mm. From the lateral beam profiles in Figure 4c, the −3 dB lateral beam widths of the concave and the double-focused transducers measured 158 μm and 95 μm; their −3 dB depth of focus (DOF) were 2.19 mm and 0.803 mm, respectively, as shown in Figure 4d. This means that the focal area generated by the double-focused transducer was reduced by approximately 80%. Note that the maximum pressure of the double-focused transducer was 108 kPa whereas that of the concave transducer was 84.8 kPa when a 7-MHz one-cycle sine wave with 1 V was used to excite the transducers.

### 2.2. 1-3 Piezo-Composite Design

The press-focusing technique was chosen as a fabrication method for a concave transducer to conduct initial focusing in this study [15]. The press-focusing process generally causes a piezoelectric material to easily crack. To prevent this, we employed a 1-3 piezo-composite as an active material so as to take advantage of high flexibility of the active layer [12,19]. In addition, a 1-3 piezo-composite is beneficial to improve transmission efficiency due to both high electromechanical coupling coefficient and reduction of the acoustic impedance of an active layer. Especially, since acoustic matching layers are not generally used in a HIFU transducer, lowering the acoustic impedance of an active layer results in reducing the difference in acoustic impedance between the active layer and the tissue (or water), thus increasing transmission efficiency.

In the piezo-composite design, ceramic pillar width, kerf, and composite thickness should be determined with a given piezoelectric material and kerf filler. The simplest and reasonably accurate method is a 1-D Krimholtz, Leedom and Matthei (KLM) equivalent circuit model in conjunction with the thickness-mode oscillation in a thin piezo-composite [20,21]. The design procedure begins selecting the pillar width and kerf that determine a ceramic volume fraction. The thickness-mode oscillation model provides the material properties of a piezo-composite material, which are changed as a function of the ceramic volume fraction of the piezo-composite. Note that a volume fraction is typically selected when the electromechanical coupling coefficient of a piezo-composite becomes maximum. After that, the thickness of a piezo-composite is selected using a 1-D KLM equivalent circuit model (e.g., PiezoCAD; Sonic Concepts, Woodlinville, WA, USA) based on the material properties of the piezo-composite.

For the 1-3 peizo-composite in this study, PZT-5H (CTS3203HD, CTS Technology, Sparta, Greece) and a mixture of Epo-tek 301 (Epoxy technologies, Billerica, MA, USA) and LP-3 (Thiokol Corp., Chiba, Japan) as a kerf filler were used. The widths of kerf polymer, *d_p_*, and ceramic pillar, *d_c_*, were decided in order to avoid the first lateral mode [22], i.e.:

(11) $$d_{p}<\frac{V_{s}}{4\sqrt{2}f_{c}}$$

(12) $$d_{c}<H/2$$

where *V_s_* is the shear velocity of a kerf polymer and *f_c_* is the desired center frequency. Note that the thickness of a 1-3 piezo-composite, *H*, is determined based on PiezoCAD simulation results and should satisfy Equation (12). Otherwise, *d_p_* and *d_c_* should be changed to meet Equations (11) and (12) while maintaining the optimal volume faction. As a result, the ceramic pillar width and kerf polymer were chosen to 61 μm and 19 μm and, thus, the volume faction was 58%; the thickness of the 1-3 piezo-composite was 278 μm. Additionally, the material properties of the 1-3 piezo-composite are summarized in Table 2.

### 2.3. Fabrication

**Figure 5:**
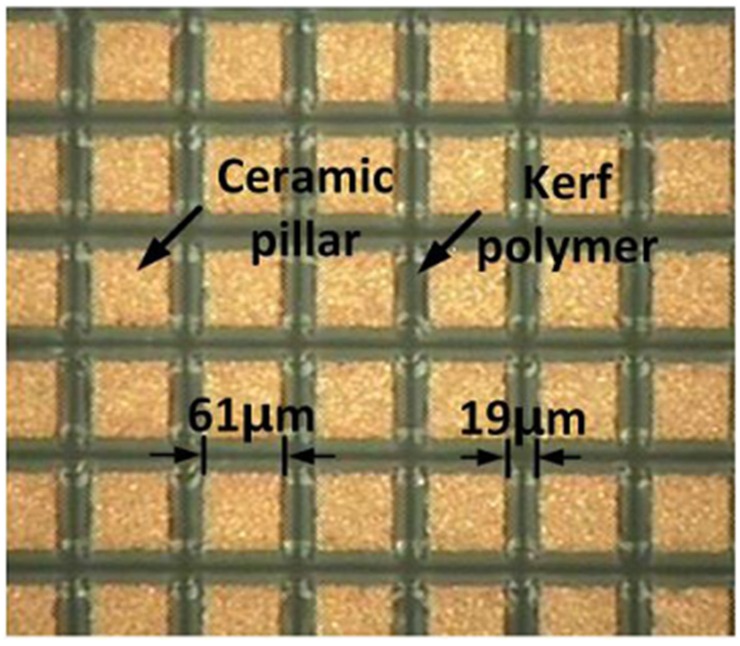
A photograph of the fabricated 1-3 piezo-composite.

The fabrication began with constructing the 1-3 piezo-compostie with a bulk piezoceramic, PZT-5H measuring 21 × 21 × 1 mm^3^. A blade (ZH05-SD2000, Disco Corp., Tokyo, Japan) with a thickness of 19 μm was used to dice the bulk piezoceramic in a dicing machine (DAD322, Disco Corp., Tokyo, Japan). The dicing along the horizontal direction was firstly conducted: a dicing depth of 350 μm and a pitch of 80 μm. Prior to dicing in the vertical direction, the separated empty space was filled with a kerf filler, i.e., the mixture of Epo-tek 301 and LP-3. After curing for 24 h at a room temperature, the second dicing was performed along the vertical direction. After the same kerf filling and curing, the complete 1-3 piezo-composite was lapped to a target thickness of 278 μm as shown in Figure 5 and shaped into circle with a 10 mm diameter. Finally, the 1-3 piezo-composite was sputtered with chrome/gold of 500/5000 Å to connect an electrode.

For press focusing, we prepared a molder made out of RTV664 (Momentive Performance Material Inc., Waterford, NY, USA), which had a hemispherical shape. The RTV molder was used for bending the composite to the target curvature. In the press-focusing procedure, the RTV molder was placed on a customized fixture and heated to 100 °C in an oven. When the temperature reached 100 °C, the fabricated 1-3 piezo-composite was put on the center of the RTV molder and pressed by a 30-mm-diameter steel ball. Once the press was finished, the cooling process was conducted at room temperature for 24 h.

To achieve the maximal transmit acoustic power, air backing was used for the double-focused transducer. For this, a brass cylinder with an inner diameter of 10 mm was filled with Epo-tek 301, which was cured for 24 h. A hole smaller than the diameter of the brass cylinder was drilled at the center of the Epo-tek 301 block. By doing so, an electrical short can be prevented because the brass cylinder played a role of electrical ground. After this, the press-focused 1-3 piezo-composite was placed onto a glass plate and the brass cylinder was laid down on the glass plate so as to place the composite in the center of the hole. The gap between the brass cylinder and the composite was filled with Epo-tek 301 to bond together. For ground connection, the front face of the composite was sputtered with Cr/Au to connect it with the brass cylinder. Also, a 160-μm wire was attached on the backside of the composite and connected to the signal line of a SMA (subminiature version A) connector.

**Figure 6:**
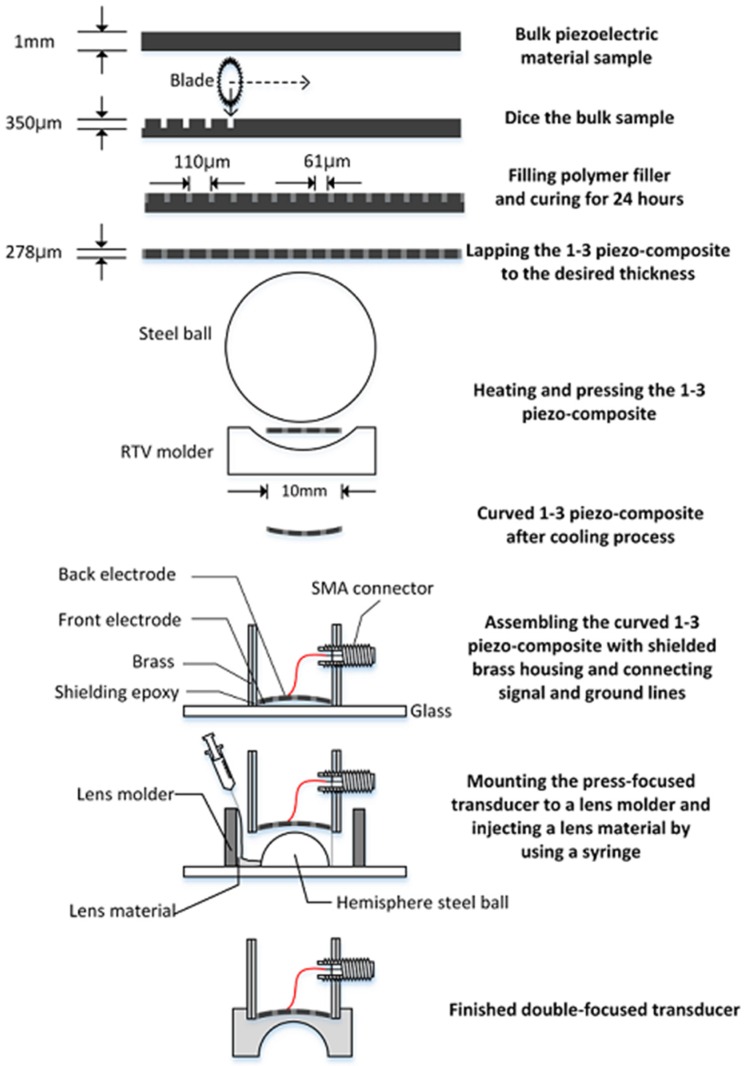
Illustration of the step-by-step fabrication process for a double-focused transducer.

**Figure 7:**
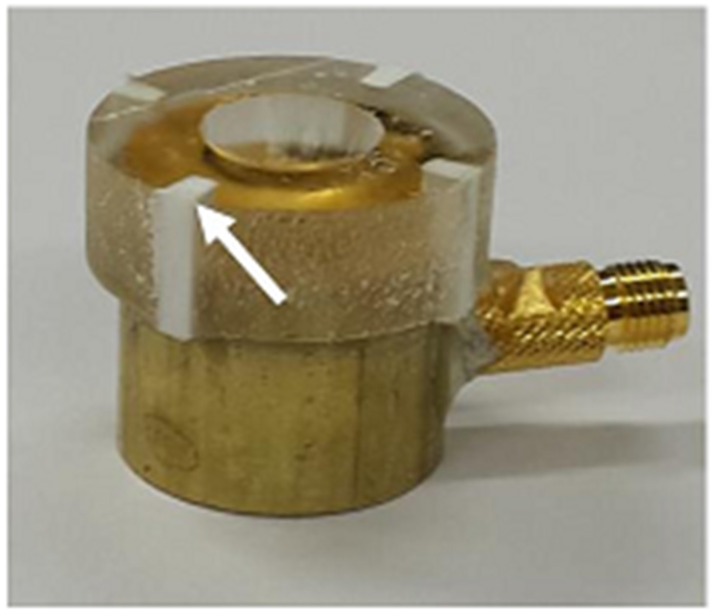
Photograph of the finished double-focused transducer. The white arrow indicates the part of an acoustic lens molder remaining after lapping. The lens molder was used to secure the maximum thickness of the lens, i.e., 6.5 mm.

For an acoustic lens, a molder was designed to guarantee the maximum thickness of the lens, i.e., L in Equation (10) (6.5 mm in this study). A 3-D printer (3DISON MULTI, ROKIT Inc., Seoul, Korea) was used to fabricate the lens molder. First, a hemispherical steel ball with a 12 mm diameter was fixed on a glass plate and the lens molder was put on the steel ball. Additionally, the press-focused transducer was fixed in the lens molder. After this, the lens material of Epo-tek 301 was cast in the lens molder and cured for 24 h at a room temperature. Finally, the lens molder was removed by lapping. Figure 6 illustrates the fabrication process. Figure 7 shows the finished double-focused transducer, in which the white arrow indicates the rest of the lens molder remaining after lapping.

## 3. Results and Discussion

**Figure 8:**
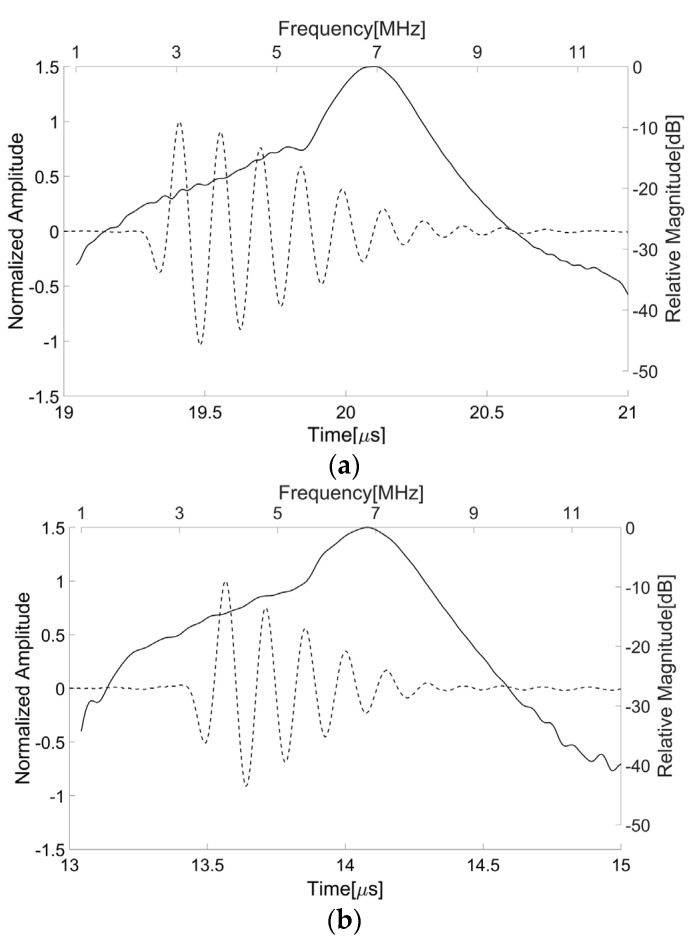
Pulse-echo waveforms and their spectra measured from the press-focused transducer before the lens casting process (**a**) and the double-focused transducer fabricated in this study (**b**). The solid line indicates the magnitude of the spectrum and the dotted line is the pulse-echo waveform.

The center frequency and fractional bandwidth were estimated by measuring the pulse-echo responses of the transducer. For this, a steel target plate was immersed in a deionized water bath and the transducer was positioned opposite the target. The distance between the target and transducer was adjusted to place the target at the focal point of the transducer. A pulser/receiver system (5800PR, Olympus, Japan) was used to excite the transducer with a transmit energy of 12.5 μJ. Note that its output electrical impedance measured 50 Ω. The received signal from the target was attenuated by 20 dB in order to avoid signal saturation in a digital oscilloscope (DPO7054, Tektronix Inc., Beaverton, OR, USA) recording the pulse-echo waveforms. Figure 8a shows the pulse-echo waveform and its spectrum measured from the press-focused transducer before the lens casting process; the peak frequency was 6.96 MHz, the center frequency was 6.88 MHz, and the −6 dB fractional bandwidth was about 1.56 MHz. Additionally, the maximum amplitude was obtained when the target was positioned at 14.8 mm away from the transducer. Note that the press-focal depth was designed to be 15 mm. As shown in Figure 8b, the double-focused transducer generated ultrasound with a peak frequency of 6.84 MHz, a center frequency of 6.77 MHz, and a −6 dB fractional bandwidth of 1.91 MHz. Its focal length measured 10.3 mm that was similar to a target focal length of 10 mm.

**Figure 9:**
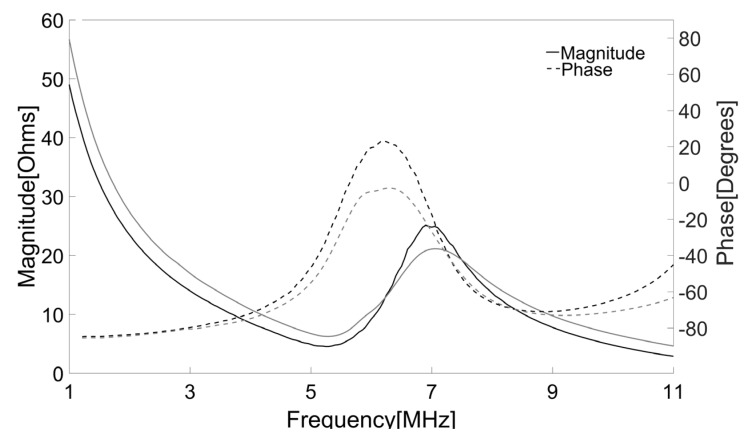
Measured electrical impedances of the press-focused (black) and double-focused (gray) transducers. The solid line represents impedance magnitude and the dotted line is phase angle.

The electrical impedance of both press-focused and double-focused transducers was measured using an impedance analyzer (HP4294A, Agilent, Santa Clara, CA, USA). Since an acoustic load affects electrical impedance and a press-focused transducer should be used in water, both transducers were immersed into the water bath for the measurement. Note that the electrical impedance of the double-focused transducer was the same regardless of the acoustic load, i.e., either water or air, because it had the lens as an acoustic load. The measurement was conducted in a frequency range from 1 MHz to 11 MHz at an increment of 0.05 MHz. As shown in Figure 9, the press-focused transducer had an impedance magnitude of 25 Ω and a phase angle of −20.6° at its peak frequency. On the other hand, those were, respectively, 20.5 Ω and −23.6° at the peak frequency in the case of the double-focused transducer.

**Figure 10:**
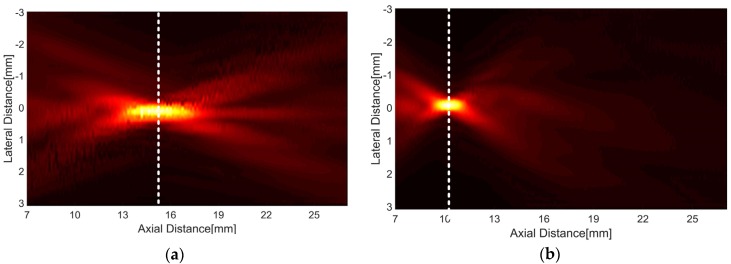
One-way radiation patterns of the press-focused transducer (**a**) and the double-focused transducer (**b**), which were measured by a hydrophone. The white dashed line indicates the depth at which the maximum pressure occurred.

To investigate the focal length, the transmit ultrasound was measured using AIMS (Acoustic Intensity Measurement System, ONDA Corporation, Sunnyvale, CA, USA) equipped with a needle hydrophone (HGL-0200). The transducer was aligned by controlling a motorized two-rotational axis against the hydrophone, horizontally. For signal generation, a function generator (AFG3232C, Tektronix Inc., Beaverton, OR, USA) in burst mode (i.e., sine wave with 1 cycle and 5 Vp-p) was used. A focal length was determined when the amplitude of the ultrasound received by the hydrophone became the maximum value. As shown in Figure 10, the measured one-way radiation patterns demonstrated that the focal length of the double-focused transducer was shorter than that of the press-focused transducer. The double-focused transducer had the peak pressure at 10.2 mm and the press-focused transducer did at 15.2 mm. This means that the focal depth of the double-focused transducer is 3.5 mm when the transducer is in contact with the skin surface because the maximum thickness of a standoff made out of the lens material is 6.5 mm. Note that the proposed transducer does not require any additional acoustic media such as water. In addition, the double-focused transducer provided the −3 dB lateral beam width and DOF of 250 μm and 1.33 mm, respectively, whereas those were 325 μm and 3.2 mm in the case of the press-focused transducer. These results agree well with the theoretical values obtained from Field II simulation; the −3 dB lateral beam width and DOF were 224 μm and 1.4 mm when a concave transducer with an aperture of 10 mm in diameter and a focal length of 10 mm was simulated. Also, those were 336 μm and 3.2 mm in the case of a 15 mm focal length. Under the assumption where a focal volume is a rectangular parallelepiped and elevation beam width is equal to lateral beam width for a simple calculation, the −3, −6, −10, and −12 dB focal volumes were obtained to be 0.08, 0.26, 0.50, and 0.67 mm^3^, respectively; those of the press-focused transducer were 0.34, 1.03, 3.00, and 4.40 mm^3^. This implies that the double-focused transducer has the beneficial ability to induce coagulative necrosis in a small volume (e.g., 1 mm^3^) because cavitation occurring during HIFU treatment possibly results in lesion enlargement [13,23,24]. In Figure 10, the white dashed lines indicate the depth at which the maximum pressure occurred: 15.2 mm in the case of the press-focused transducer and 10.2 mm in the double-focused one. These experimental results suitably demonstrated that the proposed double-focusing technique is capable of building a focal point at the desired location without reducing aperture and, thus, achieving tighter focusing of ultrasound energy.

The proposed method has adverse factors that result in an ultrasound energy loss. The first factor is signal attenuation occurring inside the acoustic lens; the attenuation coefficient of Epo-tek 301 is 3.6 dB/mm at 3.5 MHz, which is larger than that of water. The effective lens thickness ranges from 500 to 670 μm in the current design although the standoff made of the lens material has a thickness of 6.5 mm. Thus, it is computed that an energy loss in the lens is maximally 4.8 dB. Another adverse factor is ultrasound reflection from the boundaries between the piezo-composite and lens and between the lens and skin. The conventional concave transducer also suffers from the ultrasound reflection. In general, since therapeutic transducers do not have acoustic matching layers, a high ultrasound energy reflection occurs in the boundary between piezoelectric material and water. In fact, the amount of the energy reflected from the boundary is larger than that from the boundary between a piezoelectric material and an acoustic lens, i.e., Epo-tek 301 in our case; the reflection coefficient of the former case is computed to be −0.83 and that of the latter is −0.69 when the acoustic impedances of the piezo-composite, the Epo-tek 301, and water (or skin) are 16.59, 3.05, and 1.5 MRayls [25]. Note that an incident angle should be zero in this calculation. In the double-focused transducer, another reflection occurs in the boundary between the lens and water and the reflection coefficient is −0.34 if an incident angle is ignored. Therefore, the final transmission coefficient of the proposed method is 0.20 (i.e., (1 − 0.69) × (1 − 0.34)), whereas that of the conventional press-focused transducer is 0.17 (i.e., 1 − 0.83) even under the condition where the signal reflection from the acoustic membrane in a HIFU cartridge is not considered in this calculation. This indicates that the proposed method is capable of delivering higher ultrasound energy than the conventional one. However, since the maximum incident angle at the lens-skin interface in the case of the proposed method is larger than that at the water-skin interface in the conventional one, the ultrasound reflection may be more critical for the proposed method than the conventional one.

**Figure 11:**
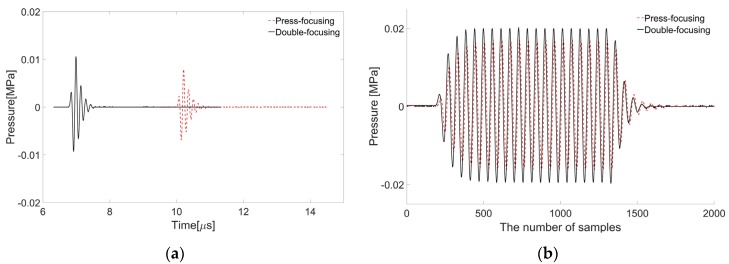
Waveforms measured by a hydrophone after delivering a 7-MHz sine wave with one cycle (**a**) and with 20 cycles (**b**) to each transducer. The maximum magnitude appeared at a depth of 15.2 mm in the case of the press-focused transducer (dotted line) and 10.2 mm in the double-focused transducer (solid line).

In spite of these adverse factors, as a result, the double-focused transducer is capable of delivering higher ultrasound energy to a treatment area than the conventional one; at the focal depth, the maximum pressure of the double-focused transducer was measured to be 19.9 kPa, whereas that of the press-focused transducer was 14.8 kPa after the signal generation of a 7-MHz, 5-Vp-p sine wave with one cycle as shown in Figure 11a. In the case of a 7-MHz, 5-Vp-p sine wave with 20 cycles, the maximum pressure was increased to 40 kPa in the case of the double-focused transducer and 33.2 kPa in its counterpart as shown in Figure 11b. This means that the tight focus achieved by the double-focused transducer is beneficial to increase ultrasound energy in a desired focal area without increasing aperture size, which was confirmed by the simulation and experimental results.

## 4. Conclusions 

In this paper, we have proposed the double-focusing method to achieve the F-number of unity or less. In the proposed method, the aperture of an ultrasound transducer is spherically shaped for initial focusing and an acoustic lens is used to finally focus ultrasound on target depth. To verify the advantage of the proposed method, we designed and fabricated a double-focused ultrasound transducer with the F-number of unity. The experimental results demonstrated that the proposed double focusing method is capable of reducing the focal length from 15.2 mm to 10.2 mm while maintaining the aperture size to be 10 mm in diameter. Therefore, the F-number of the double-focused transducer was 1.02 whereas that of the press-focused transducer was 1.52.

In addition to skin disease treatment, we believe that another potential application of the proposed method is to generate a microbeam for acoustic tweezers [26,27,28]. This is because the acoustic tweezers also require the tight focus of ultrasound. In other words, the focusing tightness determines a trapping force and, thus, it is desirable that the F-number is less than unity [29]. Since it is difficult to use either press- or lens-focusing techniques in order to fabricate an ultrasound transducer with the F-number less than unity, the proposed double-focusing method is viable for this purpose.

## Figures and Tables

**Table 1:** Geometric parameters and sound velocities of the media used for the design of the double-focused transducer.

Parameters	Value
Aperture size (mm)	10
Press-focusing focal length (mm)	15
Max. lens thickness (mm)	0.67
Standoff made of the lens material (mm)	6.5
Radius of lens curvature (mm)	6
Double-focusing focal length (mm)	10
Velocity of water (m/s)	1480
Velocity of lens (m/s)	2650

**Table 2:** Material properties of ceramic, kerf filler, lens, and 1-3 piezo-composite used for the double-focused transducer.

Parameters	Ceramic (CTS3203HD)	Kerf Filler (Epo-tek301 + LP3)	Lens (Epo-tek301)	1-3 Piezo-CompoSite
Ceramic Volume fraction (%)				50
Density (kg/m^3^)	7820	1163	1150	4325
Longitudinal velocity (m/s)	4732	2635	2650	3835
Acoustic Impedance (Mrayls)	37.0	3.06	3.05	16.59
Clamped dielectric constant	1200			741.4
Coupling coefficient	0.53			0.69
